# Breast cancer liver metastases and the impact of receptor expression on survival

**DOI:** 10.1007/s10585-025-10387-6

**Published:** 2026-01-09

**Authors:** Ellen Hansson, Marcus Sundén, Charlotta Wadsten, Gunilla Rask, Anne Andersson, Malin Sund, Oskar Hemmingsson

**Affiliations:** 1 Department of Diagnostics and Intervention, Surgery, Umeå University, 901 85 Umeå, Sweden; 2 Department of Diagnostics and Intervention, Oncology Umeå University, 901 85 Umeå, Sweden; 3 Department of Surgery, University of Helsinki and Helsinki University Hospital, 002 90 Helsinki, Finland

**Keywords:** Breast cancer, Breast cancer liver metastases, Estrogen (ER), Human epidermal growth factor receptor 2 (HER2), Progesterone (PgR), Receptor conversion

## Abstract

The aim was to determine the frequency of altered receptor expression between primary breast cancer and liver metastases, and to examine the impact of receptor expression on survival. The conversion frequency of estrogen- (ER), progesterone- (PgR) and human epidermal growth factor receptor 2 (HER2) was investigated. The prognostic value of the receptor status in the primary tumor versus the metastases was estimated. Data on a population-based regional cohort of 7292 breast cancer patients from 2009 to 2018 were collected from the National Breast Cancer Register. Biomarker expression and intrinsic subtype was studied among those who developed liver metastases with available histopathological records. The study included 311 patients with liver metastases. Conversion of ER, PgR and HER2 occurred in 16%, 47% and 12% of patients, respectively. The subtype converted in 26%. HER2 amplification in the primary tumor or metastases was associated with improved survival. Positive ER and PgR in breast cancer and positive ER in liver metastases were beneficial for survival. A combined primary tumor and metastasis receptor evaluation had the highest prognostic value. Receptor conversion from primary tumor to liver metastases is common. HER2 amplification and positive ER or PgR are associated with improved survival. Accordingly, luminal HER2 positive tumors have improved survival compared to other intrinsic subtypes. To personalize treatment for each patient, a liver biopsy is warranted at diagnosis of breast cancer liver metastases.

## Introduction

Breast cancer is the most common cancer among women [1]. Prognosis has improved significantly in recent years due to early detection by screening and novel systemic treatments [2]. However, advanced breast cancer with distant metastases remains incurable [3]. Prevalent sites of distant metastases include bone, lung and liver [4], although the incidence at each site is uncertain. Among patients with advanced breast cancer 7–70% develop liver metastases, the incidence increasing with disease duration [5–7]. Whilst bone metastases may remain stable for years with palliative treatment, liver metastases frequently result in early death. The mean survival after diagnosis of liver metastasis is 2–3 years with treatment and less than a year without treatment [6, 8].

Breast cancer is conventionally staged based on the tumor size (T), nodal involvement (N) and development of distant metastases (M), resulting in a TNM-stage [10]. The intrinsic subtypes of breast cancer, luminal A-like, luminal B-like, HER2-enriched and basal-like, are based on gene expression by PAM50 analysis [11]. The St Gallen Classification is a consensus-based subtype proxy that involves immunohistochemistry for expression of human epidermal growth factor receptor 2 (HER2); progesterone receptor (PgR) and estrogen receptor (ER). It also includes proliferation index by expression of antigen Kiel 67 (Ki-67). The classification system combines these factors into five subtypes: luminal A-like; luminal B-like; luminal HER2 positive; non-luminal HER2 positive and triple negative breast cancer [12, 13]. In the Swedish national guidelines for breast cancer Nottingham histologic grade is also included for subtyping, distinguishing luminal A-like and luminal B-like tumors from each other [10].

The classification holds predictive and prognostic value for patients diagnosed with breast cancer and is crucial for choice of therapy [13, 14]. Preserved expression of ER or PgR is associated with improved survival, and the effect of endocrine therapy correlates significantly to ER positivity [15, 16]. HER2 amplification leads to more aggressive disease [17], however it is also predictive for response to targeted anti-HER2 therapies [18].

Receptor conversion in breast cancer refers to a change in receptor status in cancer cells during disease development and occurs frequently from primary tumor to distant metastases. This potentially indicates a need to redirect tailored treatment [19–22]. We recently [23] reported that conversion of receptor status from primary tumor to liver metastases is common, and that HER2 amplification appears to be a positive prognostic factor in these patients. Yet, these results have not been validated in a secondary cohort and there is limited knowledge about receptor conversion in breast cancer liver metastases specifically.

Here, we analyze the frequency of receptor conversion between the primary tumor and corresponding liver metastases in a regional population-based cohort. We further investigate whether the receptor status of the metastasis is a superior prognostic biomarker compared to the receptor status of the primary tumor. Finally, we examine if a specific receptor expression is associated with increased survival, and if HER2 amplification [23] can be validated as a positive prognostic factor.

## Materials and methods

### Study population

This study is based on data from the Swedish National breast cancer registry (NBCR). NBCR is linked to the population register and the national cancer registry, which includes 99% of all breast cancer patients in Sweden [10]. In this population-based study, 7292 patients were included during 2009–2018 from a population of approximately 900.000 inhabitants in the counties of Västernorrland, Jämtland, Västerbotten and Norrbotten. The cohort is called the North Sweden breast cancer cohort (NSBCC).

The dataset includes information about age at BC diagnosis, date of diagnosis, vital status (alive/dead), date of death, TNM-stage, NHG, cell proliferation rate by Ki-67 and receptor expression status in the primary tumor. NBCR data were complemented by individual case report forms to add information about metastases since these are not well registered in the NBCR. Liver metastases were identified by review of radiology records in medical charts. In those patients, histopathological reports were analyzed to register receptor status and proliferation rate in the metastases. Number and size of liver metastases were retrieved from medical records. Oligometastases, were defined as 1–5 liver metastases [24]. Synchronic liver metastases were defined as patients who were reported as M1 in the NBCR at the time of breast cancer diagnosis and their first site of metastasis included the liver. Last dates of follow up were October 9, 2022; December 16, 2022; November 23, 2023 and January 27, 2023 for the Jämtland, Norrbotten, Västerbotten and Västernorrland regions, respectively. Vital status was assessed on August 18th 2020. In patients diagnosed with liver metastasis by August 18th 2020 or later, vital status was reassessed on December 12, 2024; December 17, 2024 and January 5, 2024 for the Västerbotten, Västernorrland and Norrbotten regions, respectively.

### Inclusion and exclusion criteria

Patients from the NSBCC who developed liver metastases were included in the study. Patients with missing histopathological records from both primary tumor and liver metastases and/or patients with bilateral tumors were excluded. Patients who moved to another county were censored after their relocation.

### Subtype, receptor expression and Ki-67

**Fig. 1:**
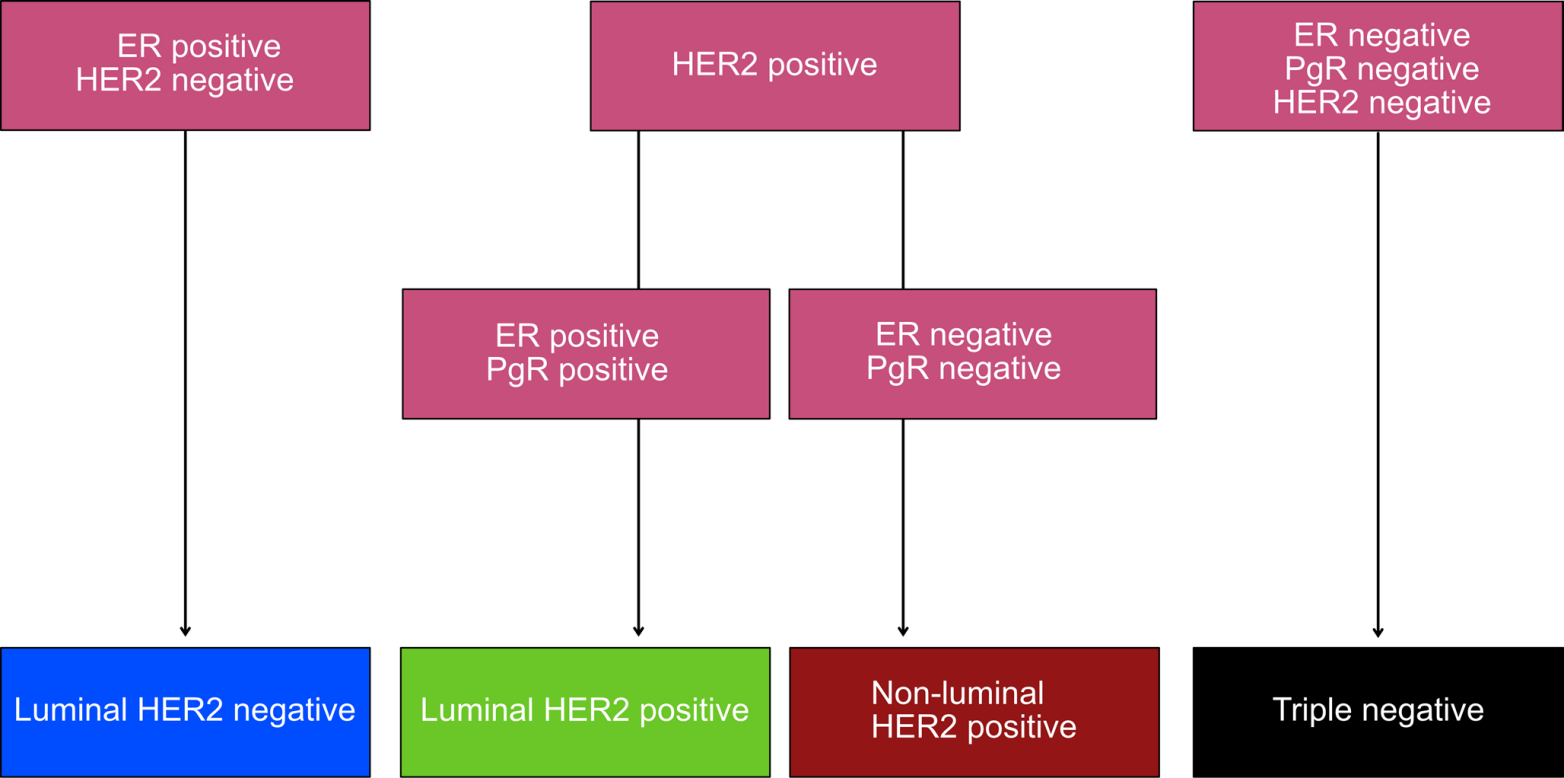
Breast cancer subtypes based on the St Gallen classification. In case of missing NHG, luminal A-like and luminal B-like cannot be distinguished from one another, resulting in a luminal HER2-negative subtype. Estrogen receptor (ER); Progesterone receptor (PgR); Human epidermal growth factor receptor (HER2)

Tumor classification was based on the St Gallen Classification and the Swedish national guidelines [10, 13]. Receptor status of ER and PgR were considered positive if the expression was stated as ≥ 10% in immunohistochemical analysis (IHC), in accordance with classification guidelines [10]. HER-2 expression was regarded positive when pathological records read 3 + in the IHC or when stated as amplified in the fluorescence or silver-enhanced in situ hybridization analysis [25]. Ki-67 was divided into low (< 10%), intermediate (10–40%) and high (> 40%) based on scoring protocols used during 2009–2018 in the regions that the cohort was based on [26]. Due to NHG not being used in liver metastases, a revised form of the Swedish classification system was applied, excluding NHG, and thereby limiting the distinction between luminal A-like and luminal B-like tumors (Fig. 1).

**Breast cancer subtype classification**

### Statistical methods

Descriptive statistics were used to present the cohort. Conversions of receptors and molecular subtypes were analyzed in openepi.com to determine a 95% confidence interval (CI) by a Wilson score for the difference in proportions [27]. For survival analyses, Cox regression was used. The PH (proportional hazards) assumption was tested by comparing estimated -ln(-ln) survival curves. The univariate Cox regression analyses included the variables presented in Table 2. Age and time from primary tumor to liver metastases was divided in two groups respectively, each divided by the median. Prognostic factors were entered into the multivariable analysis if the *p*-value was < 0.05 in the univariate analysis. To analyze survival in relation to receptor expression and subtype in breast cancer and liver metastases, Kaplan-Meier plots with log rank tests were conducted. Kaplan-Meier plots were also conducted for variables that did not meet the PH assumption, including TNM, NHG and number of liver metastases. Number of metastases were divided into two groups, 1–5 and > 5 [3].

Harrel’s C index was used to determine the prognostic value of the receptor expression in:

- Breast cancer.
- Liver metastases.
- Breast cancer and liver metastases combined.

Results with a *p*-value < 0.05 were considered statistically significant. Data analysis was conducted using IBM Corp. IBM SPSS Statistics for Mac. Version 29.0.2 (Armonk, NY: IBM Corp; 2023).

## Results

### Cohort characteristics

**Fig. 2:**
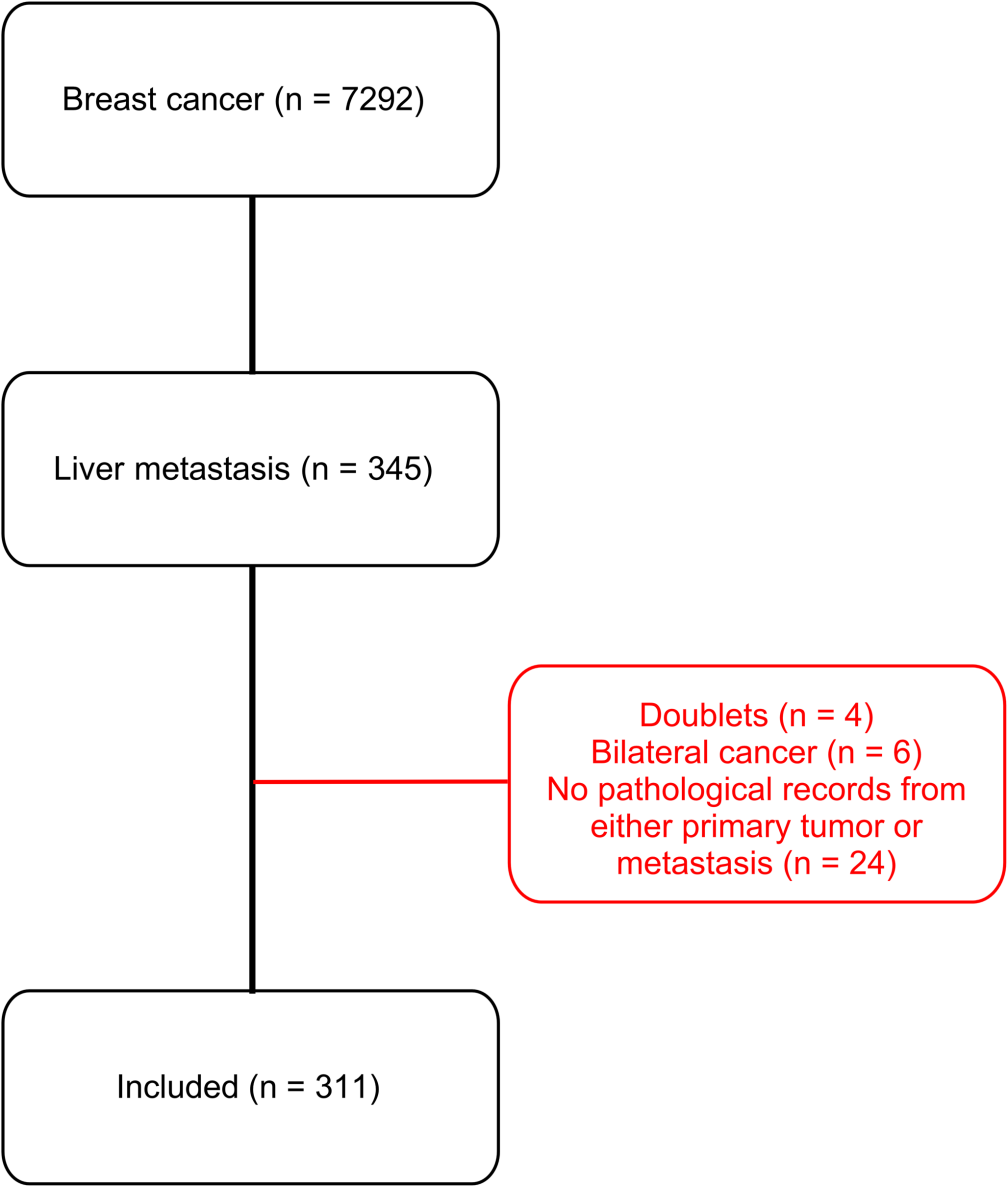
Consort diagram of inclusion

The cohort included 7292 patients diagnosed with breast cancer in the Norrbotten, Västerbotten, Västernorrland and Jämtland regions during 2009–2018. In total, 345 patients with liver metastases were identified through the review of radiology records. Of these, four patients were doublets and six patients were excluded due to bilateral cancer. Out of the remaining cohort, histopathological reports were available from the primary tumor in 309 patients, from the liver metastases in 84 patients, and from both sites in 82 patients. Patients with a report from either site were included, resulting in a total cohort of 311 patients (Fig. 2). For the receptor conversion analysis, only the patients with a report from both sites were included (*n* = 82). The mean age at breast cancer diagnosis was 62 years (95% CI 60.8–64.0) and the mean time to liver metastases was 39 months (95% CI 34.8–42.5) (Table 1). Adjuvant treatment of the primary tumor was registered in all counties except one. In the counties where treatment data were available, adjuvant treatment was overall given according to the Swedish guidelines. However, out of 36 HER2 positive patients within the counties providing data regarding treatment, 10 received adjuvant trastuzumab, seven did not receive trastuzumab and 19 had missing data. Further patient characteristics are presented in Table 1.

**Table 1:** Descriptive statistics of the cohort. Estrogen receptor (ER); progesterone receptor (PgR); human epidermal growth factor receptor 2 (HER2); Nottingham histologic grade (NHG)

Descriptive statistics	
	*n* (%)
Cohort	311
Sex (female/male)	307/4 (98.7/1.3)
Mean age at primary breast cancer (years)	62.38 95% CI 60.77–63.98
Mean age at liver metastasis (years)	65.598 95% CI 64.014–67.181
T-stage	
T0	20 (6.4)
TIS	2 (0.6)
T1	79 (25.4)
T2	131 (42.1)
T3	51 (16.4)
T4	27 (8.7)
No data	1 (0.4)
N-stage	
0	172 (55.3)
1	116 (37.3)
2	15 (4.8)
3	4 (1.3)
No data	4 (1.3)
M-stage	
0	211 (67.8)
1	63 (20.3)
No data	37 (11.9)
Subtype (primary tumor)	
Luminal HER2 negative	182 (62.5)
Luminal HER2 positive	31 (10.7)
Non-luminal HER2 positive	24 (8.2)
Triple negative	54 (18.6)
no data	20 (6.0)
ER (primary tumor)	
Negative	86 (27.6)
Positive	221 (71.1)
No data	4 (1.3)
PgR (primary tumor)	
Negative	130 (41.8)
Positive	177 (56.9)
No data	4 (1.3)
HER2 (primary tumor)	
Not amplified	241 (77.5)
Amplified	55 (17.7)
No data	15 (4.8)
Ki-67 (primary tumor)	
Low	38 (12.2)
Intermediate	52 (16.7)
High	209 (67.2)
No data	12 (3.9)
NHG	
1	9 (2.9)
2	73 (23.5)
3	133 (42.8)
No data	96 (30.8)
Extrahepatic metastases at time of liver metastasis	254 (81.7)
Surgery for liver metastasis	3 (1.3)
Mean time to liver metastasis (months)	38.663 95% CI 34.830–42.497
Adjuvant endocrine therapy	
Yes	115 (37.0)
No	46 (14.8)
No data	150 (48.2)
Adjuvant chemotherapy	
Yes	92 (29.6.8)
No	69 (22.2)
No data	150 (48.2)
Adjuvant trastuzumab	
Yes	11 (3.5)
No	149 (47.9)
No data	151 (48.6)

### Receptor and subtype conversion

**Fig. 3:**
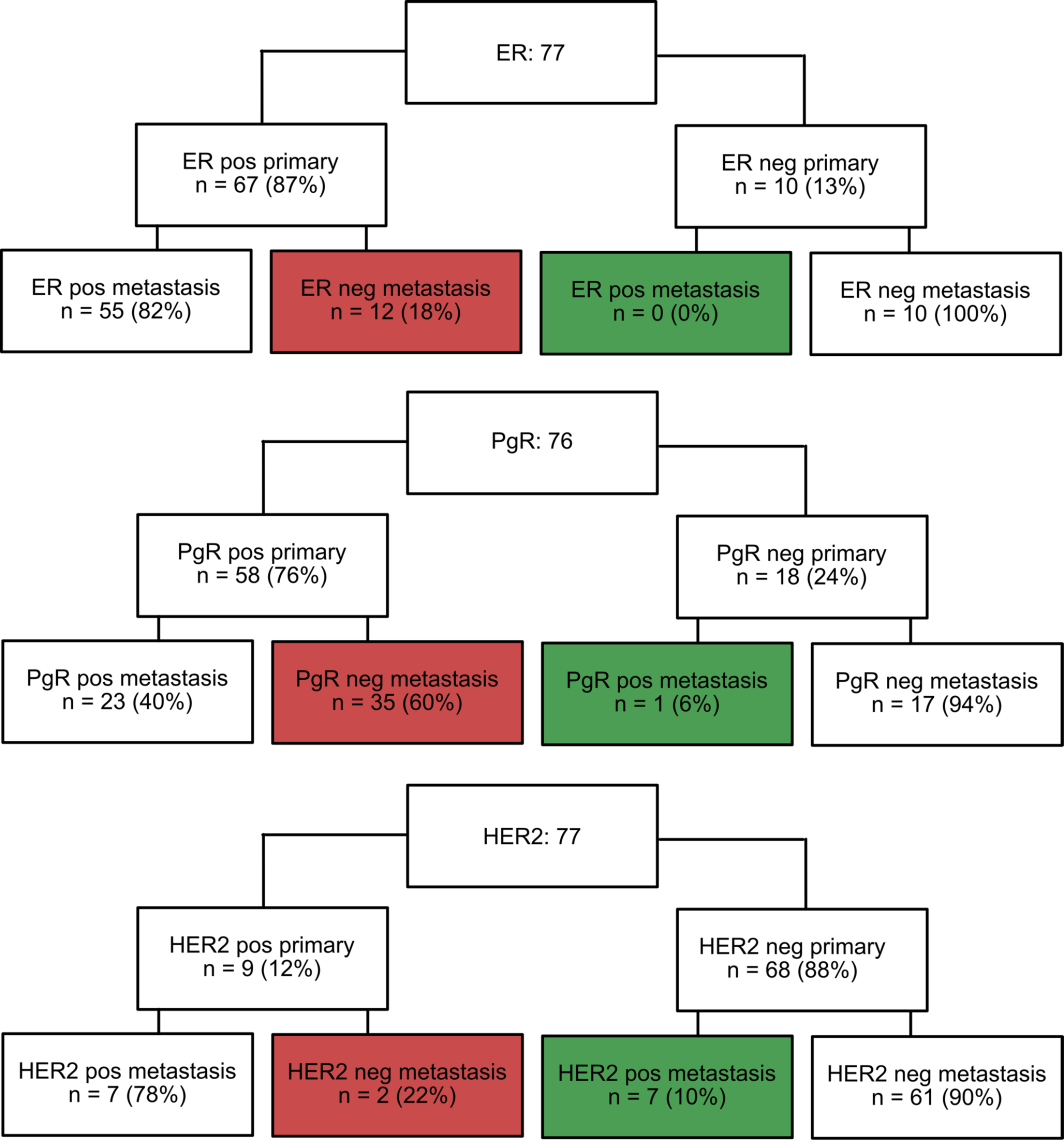
Receptor conversion from primary tumor to liver metastases. Estrogen (ER); Progesterone (PgR); Human epidermal growth factor receptor (HER2)

Patients with information on receptor status from both primary tumor and metastasis were included in the analysis of receptor and subtype conversion. We calculated the conversion rate and the status of ER, PgR and HER2 frequently changed between primary tumor and liver metastasis. ER status changed from primary tumor to metastasis in 12 of 77 patients (16%, 95% CI 0.09–0.25). Out of patients with ER positive primary breast cancer (*n* = 67), 12 were ER negative in the metastasis (Fig. 3). None of the ER negative cancers (*n* = 10), converted to an ER positive expression pattern in the metastases. PgR status converted from primary tumor to metastasis in 36 of 76 patients (47%, 95% CI 0.37–0.58). PgR positive tumors (*n* = 58) changed most frequently, with 35 turning PgR negative in the metastasis. PgR negative cancers (*n* = 18), changed to positive in one case (Fig. 3). HER2 status changed from primary tumor to metastasis in nine of 77 patients (12%, 95% CI 0.06–0.21). HER2 amplification in primary tumor (*n* = 9) converted to negative in two patients (Fig. 3). HER2 negative breast cancer (*n* = 68), changed to positive in seven metastases (Fig. 3). Out of these, two patients had IHC from the primary tumor that read 2 + but the FISH analysis classified them as not amplified. In the remaining five patients there were no detailed data on the IHC HER2 status and they were only stated as not amplified.

**Receptor conversion**

**Fig. 4:**
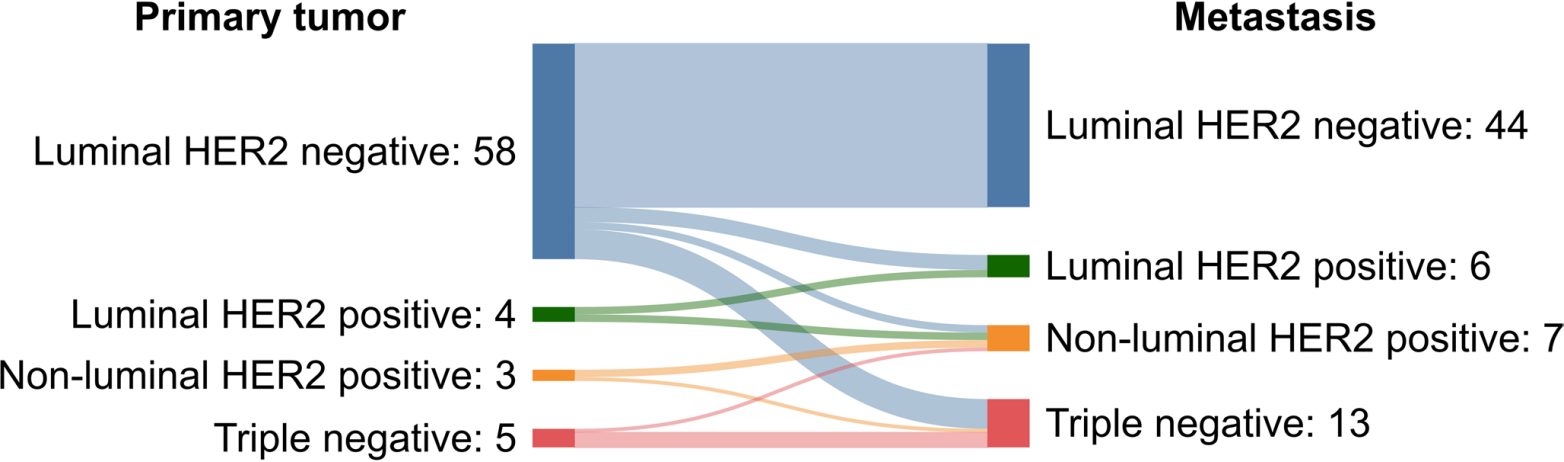
Subtype conversion from breast cancer to liver metastasis. Human epidermal growth factor receptor (HER2)

Tumor intrinsic subtypes by proxy were classified using the St Gallen Classification, but without discrimination between the luminal A-like and luminal B-like subtypes due to missing data on NHG in the liver metastases (Fig. 1). Overall, subtype changed from the primary tumor to the liver metastasis in 18 of 70 patients (25.7%, 95% CI 0.17–0.37) (Fig. 4). Luminal HER2 negative cancer (*n* = 58) changed to luminal HER2 positive in the metastases of four patients (6.9%), to non-luminal HER2 positive in two patients (3.4%) and to triple negative in eight patients (13.8%) (Fig. 4). Luminal HER2 positive breast cancer (*n* = 4), converted to non-luminal HER2 positive metastases in two patients (50.0%) (Fig. 4). Non-luminal HER2 positive tumors (*n* = 3), converted to triple negative in one patient (33.3%) (Fig. 4). Finally, triple negative primary tumor (*n* = 5), changed into non-luminal HER2 positive metastasis in one patient (20.0%) (Fig. 4).

**Subtype conversion**

### Survival analysis and prognostic value

Mean survival time from primary breast cancer diagnosis and liver metastasis diagnosis was 61 months (95% CI 55.5–66.8) and 20 months (95% CI 15.6–24.6), respectively. For survival analyses, time from liver metastasis diagnosis to death or end of the study was used. The survival analysis was conducted using Cox regression analyses and Kaplan-Meier plots. The proportional hazards assumption was overall tested successfully for the Cox regression analysis comparing estimated -ln(-ln) survival curves [28]. Biopsy of liver metastasis, TNM-status and NHG however had nonparallel curves and were thus excluded from the Cox regression analysis. They were instead analyzed with Kaplan-Meier plots. Neither TNM nor NHG were statistically significant risk factors for worse survival. However, patients with a biopsy from their liver metastasis had better survival during the first 4 years, compared to those without (not shown).

Univariate Cox regression analyses were carried out on receptor status and possible confounders (Table 2). In the univariate analyses, high age at liver metastasis and occurrence of extrahepatic metastases at diagnosis of liver metastases were associated with worse survival (HR 1.49; CI 1.16–1.91 and HR 1.59; CI 1.13–2.24, respectively) (Table 2). Patients with six or more liver metastases had worse survival than those with oligometastases (HR 1.37; 95% CI 1.06–1.77) (Table 2). Patients with ER and PgR negative primary tumors had increased HRs compared to patients with ER and/or PgR positive cancers (HR 1.41; CI 1.07–1.85 and HR 1.47; CI 1.14–1.89) (Table 2). There was no statistically significant association between ER and PgR status in the liver metastases and survival. On the other hand, HER2 positivity, in both the primary tumor and in liver metastases, was associated with decreased mortality compared to patients with no HER2 amplification (HR 0.44; CI 0.30–0.63 and HR 0.37; CI 0.16–0.89). The variables with a *p*-value < 0.05 were further included in the multivariable analysis (Table 2). In this analysis, HER2-status in the liver metastases was excluded since it would have limited the number of events from 206 to 46. In total, 288 patients were included in the multivariate regression analysis, which showed that higher age (HR 1.36 (1.04–1.77); 0.02) and occurrence of extrahepatic metastases was associated with worse survival (HR 1.80 (1.24–2.62); 0.002) (Table 2). Again, patients with more than five metastases had worse survival than those who had oligometastases (1–5) in the liver (HR 1.66 (1.26–2.20); <0.001). Furthermore, patients with negative PgR primary tumors had decreased survival (HR 1.75 (1.21–2.53); 0.003) and amplification of HER2 in primary tumor was associated with better survival (HR 0.40 (0.27–0.59); < 0.001) (Table 2).

**Table 2:** Survival analysis of the cohort. Estrogen (ER); progesterone (PgR); human epidermal growth factor receptor (HER2). Statistically significant variables are highlighted in bold.

Cox regression analysis		
Prognostic factor	Univariate analysis	Multivariable analysis
	HR (95% CI); *p*-value	HR (95% CI); *p*-value
Age at liver metastasis		
Below median (< 68 years)	REF: 1.00	REF: 1.00
Above median	**1.49 (1.16–1.91); 0.002**	**1.36 (1.04–1.77); 0.023**
Time to liver metastasis		
Below median (< 30 months)	REF: 1.000	
Above median	1.01 (0.78.-1.30); 0.934	
Extrahepatic metastases at breast cancer diagnosis		
No	REF: 1.000	
Yes	1.14 (0.79–1.64) 0.478	
Extrahepatic metastases at liver metastasis diagnosis		
No	REF: 1.00	REF: 1.00
Yes	**1.59 (1.13–2.24); 0.008**	**1.80 (1.24–2.62); 0.002**
Number of liver metastases		
1–5	REF: 1.00	REF: 1.00
> 5	**1.36 (1.06–1.74); 0.016**	**1.66 (1.26–2.20); <0.001**
Synchronic liver metastasis		
No	REF: 1.00	
Yes	0.82 (0.60–1.11); 0.198	
ER in primary tumor		
Negative	**1.41 (1.07–1.85); 0.014**	1.43 (0.96–2.12); 0.080
Positive	REF: 1.00	REF: 1.00
PgR in primary tumor		
Negative	**1.47 (1.14–1.89); 0.003**	**1.75 (1.21–2.53); 0.003**
Positive	REF: 1.00	REF: 1.00
HER2 in primary tumor		
Not amplified	REF: 1.00	REF: 1.00
Amplified	**0.44 (0.30–0.63); < 0.001**	**0.40 (0.27–0.59); < 0.001**
Ki-67 in primary tumor		
Low	REF: 1.00; 0.66	
Intermediate	1.02 (0.68–1.78); 0.711	
High	1.19 (0.80–1.75); 0.391	
ER in liver metastasis		
Negative	1.74 (1.00–3.05); 0.052	
Positive	REF: 1.00	
PgR in liver metastasis		
Negative	0.77 (0.43–1.37); 0.377	
Positive	REF: 1.00	
HER2 in liver metastasis		
Not amplified	REF: 1.00	
Amplified	**0.37 (0.16–0.89); 0.026**	

**Fig. 5:**
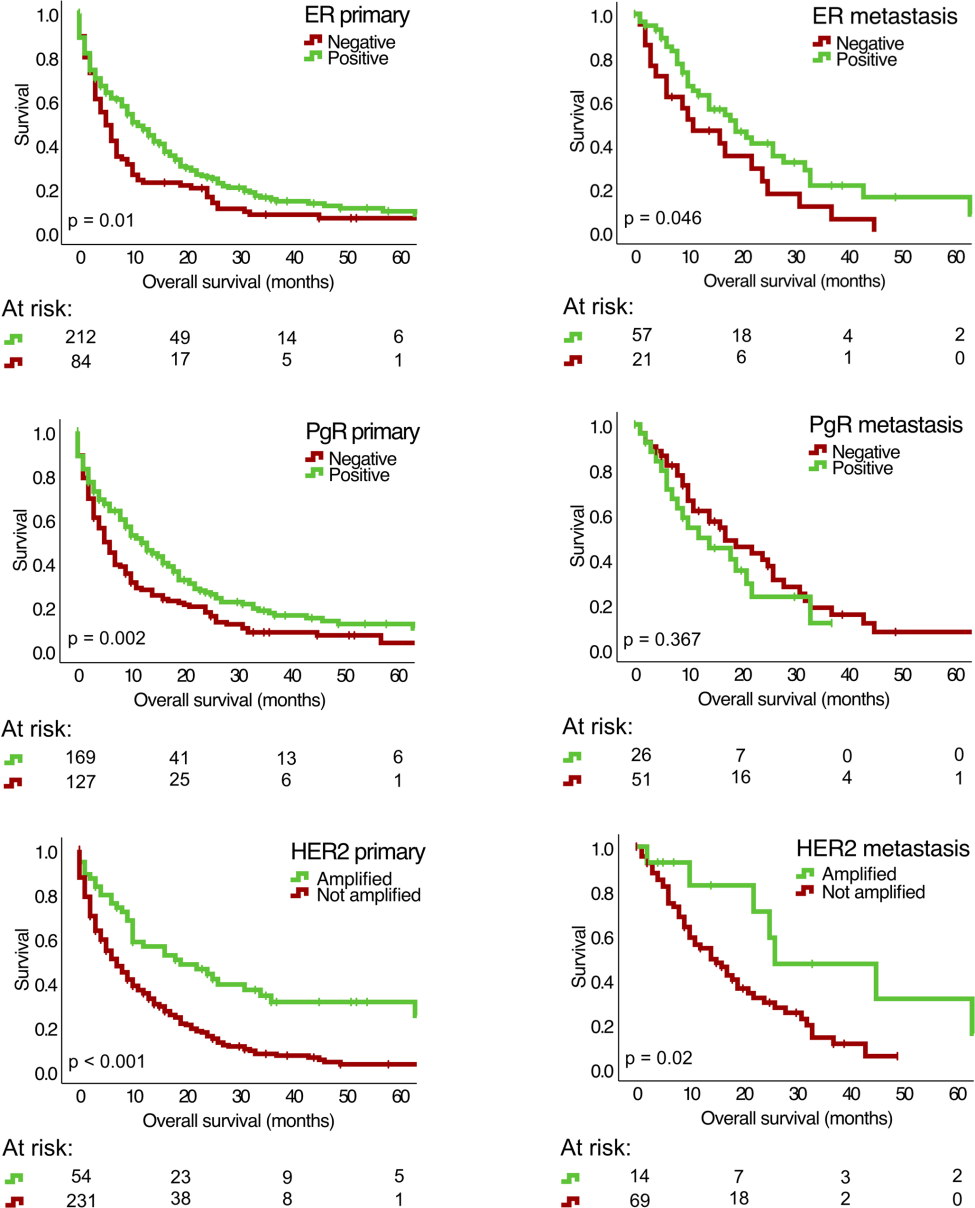
Kaplan-Meier survival plots of receptor expression in breast cancer and liver metastases. Survival time is measured from diagnosis of liver metastasis to end. Estrogen receptor (ER); Progesterone receptor (PgR); Human epidermal growth factor receptor 2 (HER2)

Kaplan-Meier plots and log rank tests were conducted comparing hormonal receptor status and subtypes of primary tumor and metastases. Positive ER status in both the primary tumor and metastases was associated with increased survival (*p* = 0.01; *p* = 0.046) (Fig. 5). Positive PgR in the primary tumor was also related to enhanced survival (*p* = 0.002), although, PgR status in the metastases did not (*p* = 0.367) (Fig. 5). Amplifications of HER2 in primary tumor and metastases were both associated with increased survival (*p* < 0.001; *p* = 0.02 respectively) (Fig. 5).

**Kaplan-Meier plots of receptor status in primary breast cancer and liver metastases**

**Fig. 6:**
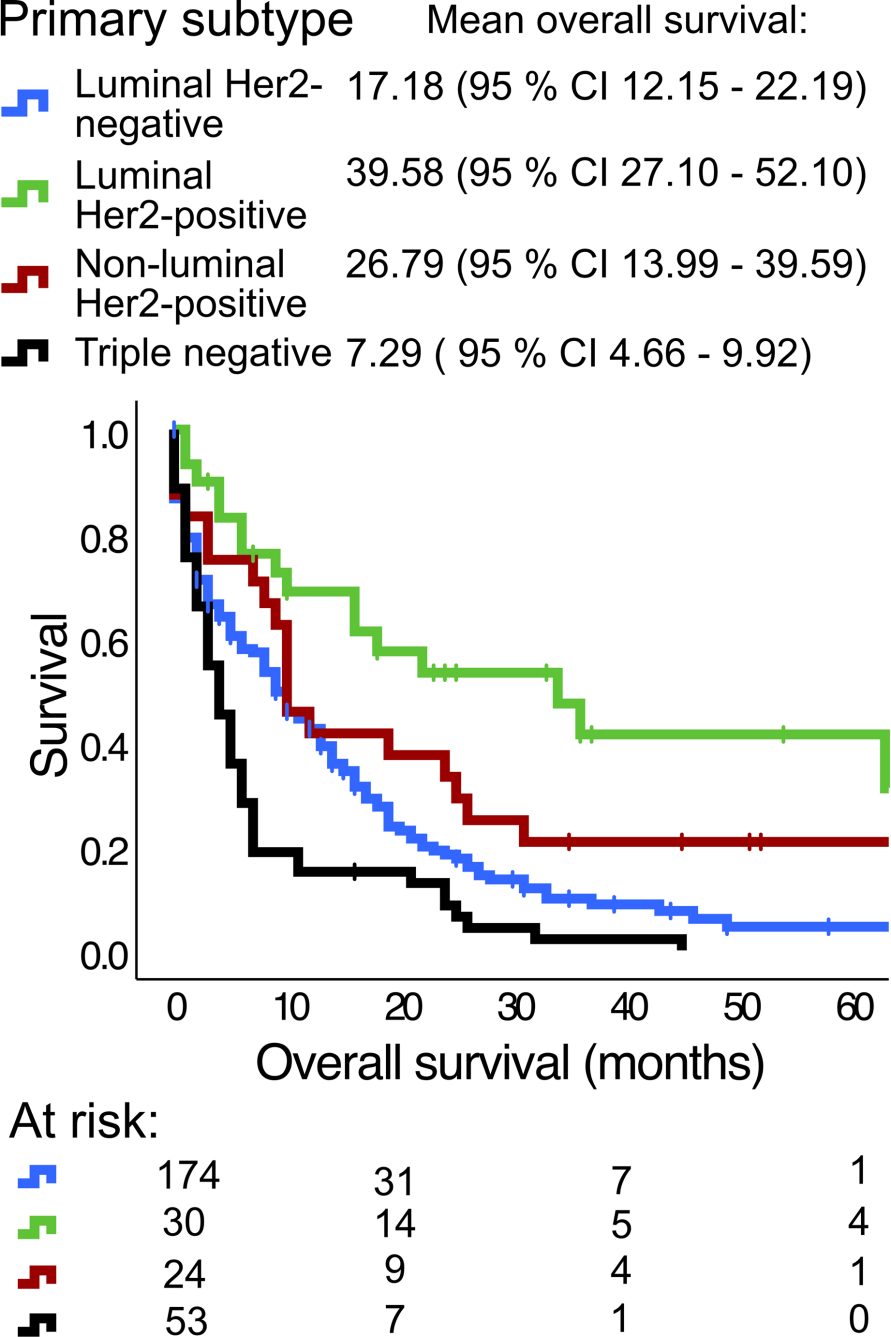
Kaplan-Meier survival plots of subtypes in breast cancer and mean overall survival in each group. Survival time is measured from diagnosis of liver metastases. Human epidermal growth factor receptor 2 (HER2)

There was a significant difference in survival time among the different breast cancer subtypes (Fig. 6). Patients with triple negative breast cancer had shorter survival than patients with other breast cancer subtypes, with luminal HER2 positive breast cancer patients having the longest survival. A Kaplan-Meier plot with a log rank test was conducted, comparing luminal HER2 negative tumors to luminal HER2 positive tumors, showing a significant longer survival for patients with luminal HER2 positive cancers. Comparing subtypes in the liver metastases showed similar results but the analysis was hampered by having only few patients in each subtype category.

**Kaplan-Meier of breast cancer subtype**

To determine the prognostic value, Harrel’s C index was calculated, including the same variables as in the multivariable Cox regression analysis. The index was calculated based on three models. The first based on receptor expression in the primary tumor, the second on receptor expression in the liver metastasis and the third based on both combined. Preferably, the index should be as close to 1 as possible. Harrel’s C index was 0.631 and 0.668, when using data from the primary tumor and liver metastases, respectively. Combining data from both gave the highest index, 0.686.

## Discussion

This study showed that conversion of ER, PgR and HER2 status is common during disease progression from primary breast cancer to breast cancer liver metastasis. This aligns with previous research [19–23] and emphasizes the aspects of tumor progression that is also seen in ductal adenocarcinoma of pancreas [29] and colorectal cancer [30]. The receptor expression holds prognostic value in patients developing liver metastases. Receptor conversion occurred mainly as loss of PgR or ER expression, hence leading to breast cancer group with worse prognosis. HER2 changed from negative to positive in 10% of patients, and in this study, HER2 amplification in both primary tumor and liver metastases was associated with a more beneficial prognosis. For molecular subtyping, we applied a classification system that most closely corresponded to the one in use in Sweden during the study period. Subtype conversion occurred frequently within the study cohort in line with the described receptor conversion. No distinction between luminal A-like and luminal B-like tumors could be made, likely leading to an underestimated number of subtype conversions. These were thus combined into luminal HER2 negative tumors, that most frequently turned into triple negative in the liver metastases. However, 7% of the patients with luminal HER2 negative subtype developed amplification of HER2 in the liver metastases, leading to an opportunity of trastuzumab treatment in those patients. The limited number of patients with luminal HER2 positive, non-luminal HER2 positive and triple negative subtypes made it difficult to come to any reliable conclusions regarding those patients. The results regarding receptor and subtype conversion mainly align with the results from Sundén et al. [23].

In the present material, positive ER, PgR or HER2 expression in primary tumor, and positive ER or HER2 expression in liver metastasis were beneficial for overall survival compared to those with negative receptors. These results show similar patterns to Sundén et al. [23], although expression of PgR in the metastases in this study and ER expression in the metastases in Sundén et al. [23] did not reach statistical significance. While PgR and ER expression have long been recognized as prognostic factors, the role of HER2 amplification remains more uncertain. The previous study from our group [23] indicated that positive HER2 is a favourable prognostic marker in patients with liver metastases, a finding validated by this research.

Given the oncogenic nature of HER2 amplification, it stands to reason that therapies targeting this receptor, may confer significant advantages in the management of liver metastases. However, earlier research has found HER2 positive primary tumors to be a risk factor for developing liver metastases in the first place, which is a prognostic disadvantage compared to other common metastatic sites, such as bone [31]. Hence, although HER2 amplification may be beneficial in patients diagnosed with breast cancer liver metastases, the role of HER2 in development of liver metastasis remains to be explored.

Neither NHG nor TNM at diagnosis had any impact on survival in the studied material. Arguably, a higher NHG and a higher TNM, with nodal or even metastatic spread at diagnosis, should generally entail a more aggressive cancer. Hence, the lack of impact on survival observed here is perplexing. This may be due to selection of patients with liver metastases and analysis of survival after diagnosis of those. There were 20 patients that were classified as T0 and two as carcinoma in situ in the primary tumor. This is perplexing, given that they are not invasive and thus should not be able to metastasize. *Some of these may be explained by diagnosis of a breast cancer metastasis on a biopsy without finding of a primary tumor. It could also be occult breast cancer. One additional explanation could be misclassification in NBCR.*

Patients with occurrence of extrahepatic metastases at diagnosis of liver metastasis had a decreased overall survival. One possible explanation is that breast cancer patients with isolated liver metastases, have a lower tumor burden and a better performance score thus enabling more intense systemic treatment when compared to patients with generalized cancer spread. This also implies that these patients may benefit from local therapies.

One limitation to this study is that receptor expression in the liver metastases was not included in the multivariable analysis due to a limited number of events. Further, there was missing data regarding systemic therapy given at diagnosis of breast cancer, mainly regarding frequency and doses given. However, when comparing the number of ER and PgR positive patients and the number of patients receiving endocrine therapy, they are approximately equal. During the study period treatment with trastuzumab was well established in all four regions. The proportion of HER2 positive patients not receiving trastuzumab was slightly more extensive than patients not receiving endocrine therapy. This is most likely due to low performance status or high age. A big proportion of patients however had missing data regarding medical treatment, making it difficult to draw any conclusions. In this study, 341 patients developed liver metastases, of whom 311 had biopsy from either primary tumor or metastasis. Patients lacking these were excluded from all analyses, which could have impacted the mean survival. These patients likely had synchronous other distant metastases and were ineligible for primary tumor surgery. Biopsy of suspected liver metastases is recommended for diagnosis confirmation in international guidelines [3, 32]. The number of patients undergoing a liver biopsy with receptor status analyses was lower than expected in this material, given that the proportion of patients who underwent liver biopsy in Sundén et al. [23] was about twice as high. However, patients who had a liver biopsy likely had better performance state than those without tissue confirmation, as a biopsy is generally feasible for patients in a better condition. Indeed, biopsy was associated with improved survival (not shown). Besides the somewhat low proportion of patients that underwent liver biopsy and the risk of selection bias, the distribution regarding breast cancer subtype and receptor expression in both primary tumor and metastases in this population-based cohort is highly representative for the breast cancer population overall.

The difference in prognostic value of receptors status between primary tumor and liver metastasis is marginal and does not pose enough evidence to say that either one is superior to the other. However, as shown here, the value is most accurate when combining receptor status from both the primary tumor and liver metastases in the Harrel’s C index. Hence, there is reasonable to suggest that receptor expression in liver metastases contributes to an estimation of prognosis in breast cancer patients. In Sundén et al. [23] the prognostic value was also most accurate when data was combined from the primary tumor and liver metastases. Due to the low number of defined cases within each subtype in liver metastases, Harrel’s C index could not be calculated for intrinsic breast cancer subtypes.

In conclusion, in patients who develop breast cancer liver metastasis, HER2 amplification in both the primary tumor and the metastasis is associated with improved survival. Further, that positive PgR in the primary tumor and positive ER in both primary tumor and liver metastasis are favourable prognostic biomarkers. Accordingly, patients with luminal HER2 positive breast cancer have improved survival compared to patients with other subtypes. The prognostic value is most accurate when combining information about receptor expression status from both the primary breast cancer and the metastasis. Receptor and subtype conversion from primary tumor to liver metastasis is common and can lead to an opportunity to redirect treatment. Thus, a liver biopsy with receptor analysis is warranted at diagnosis of liver metastasis.

## Data Availability

The datasets generated during and/or analyzed during the current study are not publicly available due to patient confidentiality.
