# Correction: Breast cancer liver metastases and the impact of receptor expression on survival

**DOI:** 10.1007/s10585-026-10425-x

**Published:** 2026-08-18

**Authors:** Ellen Hansson, Marcus Sundén, Charlotta Wadsten, Gunilla Rask, Anne Andersson, Malin Sund, Oskar Hemmingsson

**Affiliations:** 1 Department of Diagnostics and Intervention, Surgery, Umeå University, 901 85 Umeå, Sweden; 2 Department of Diagnostics and Intervention, Oncology Umeå University, 901 85 Umeå, Sweden; 3 Department of Surgery, University of Helsinki and Helsinki University Hospital, 002 90 Helsinki, Finland

**Correction to**: Clinical & Experimental Metastasis 43:8 (2026) 10.1007/s10585-025-10387-6.

In this article,

Ellen Hansson and Marcus Sundén shared first authorship! Not Malin and Oskar Incorrect in Pubmed.

The below mentioned reference should be deleted:

Harrell FE Jr, Lee KL, Califf RM, Pryor DB, Rosati RA. Regres sion modelling strategies for improved prognostic prediction. Stat Med. 1984 Apr-Jun;3(2):143–52. 10.1002/sim.4780030207. PMID: 6,463,451.

The author Gunilla Rask should also have affiliation as mentioned below:

Department of Surgery, University of Helsinki and Helsinki University Hospital, 002 90 Helsinki, Finland.

The original article has been corrected.

